# Workplace social support in job satisfaction among veterans with posttraumatic stress symptoms: A preliminary correlational study

**DOI:** 10.1371/journal.pone.0181344

**Published:** 2017-08-04

**Authors:** J. I. Harris, Thad Q. Strom, Amanda G. Ferrier-Auerbach, Matthew E. Kaler, Lucas P. Hansen, Christopher R. Erbes

**Affiliations:** 1 Minneapolis VA Health Care System, Minneapolis, Minnesota, United States of America; 2 University of Minnesota Department of Psychiatry, Minneapolis, Minnesota, United States of America; 3 Center for Chronic Disease Outcomes Research, Minneapolis, Minnesota, United States of America; Hunter Holmes McGuire VA Medical Center, UNITED STATES

## Abstract

For Veterans managing PTSD symptoms, returning to vocational functioning is often challenging; identifying modifiable variables that can contribute to positive vocational adjustment is critical to improved vocational rehabilitation services. Workplace social support has proven to be important in vocational adjustment in both general population and vocational rehabilitation samples, but this area of inquiry has received little attention among Veterans with PTSD symptoms. In this small correlational study, employed Veterans (N = 63) presenting for outpatient PTSD treatment at a VA Health Care System completed surveys assessing demographic variables, PTSD symptoms, workplace social support, and job satisfaction. Workplace social support contributed to the prediction of job satisfaction. It is of note that workplace social support predicted a larger proportion of the variance in employment satisfaction than PTSD symptoms. Further research on workplace social support as a vocational rehabilitation resource for Veterans with PTSD is indicated.

## Introduction

Over half of the two billion dollars of our nation’s annual costs attributable to PTSD are related to loss of work productivity [1]. Successful rehabilitation from PTSD involves meaningful participation in, functioning, and satisfaction with major life roles. This can include restoring the capacity to work, which supports social role functioning and socioeconomic resources for the Veteran and his or her family, as well as community reintegration [2–4]. Enhancing the effectiveness of vocational rehabilitation services for Veterans with PTSD may improve quality of life for Veterans and reduce costs to our nation.

Veterans managing PTSD are less likely than those without PTSD to be employed, especially as symptom levels become more severe [5] or are related specifically to combat [6]. These Veterans report more work limitations, poorer work functioning, and lower levels of job satisfaction [2, 7–11]. Working Veterans managing PTSD report missing more days of work due to symptoms [8,10,12], deteriorating work performance over time [2], and low levels of social support from others at work [8]. Clearly, exploration of additional support for vocational functioning among Veterans with PTSD is indicated.

The Job Demands and Resources model (JD-R; [13–15]) provides a useful framework for conceptualizing workplace functioning among people managing PTSD. The JD-R focuses on the balance of work stressors (physical demands, time limitations, problem-solving, emotional stress, etc.) and work resources (training, infrastructure/tools, supervisory consultation and support, task support, value on work content, social support, etc.) as determinants of vocational adjustment, job satisfaction, and work performance [13, 16]. As the magnitude of work stressors (job demands) comes to exceed available work resources, vocational adjustment, job satisfaction, and performance will suffer. Managing PTSD at work can be seen as a significant job stressor because it requires coping effectively with symptoms that can interfere with other task and interpersonal demands to function in the workplace. For example, symptoms of PTSD include irritability, difficulty concentrating, and hypervigilance [17], all of which can interfere with a person’s ability to remain focused at work and to interact appropriately with co-workers or supervisors. The JD-R model [13, 16] indicates that increasing resources available to buffer work stresses (demands) can improve vocational adjustment and job satisfaction. Exploring avenues for increasing resources in the work environment may be key to enhancing rehabilitation outcomes for individuals with PTSD.

Access to a large and diverse social support network is negatively correlated with PTSD among civilian and military trauma survivors [18, 19]. While the direction of causation is unclear, workplace social support may be a modifiable, environmental variable that could enhance vocational functioning for Veterans managing PTSD. In the general population, workplace social support has been identified as a potent predictor of both job satisfaction and job tenure [20, 21]. Support from supervisors and colleagues are associated with better work functioning [22]. In a military sample, perceived support from other members of one’s unit predicted better morale and work engagement [23]. Workplace social support buffers the effects of work stressors and inhibits burnout [24, 25].

Among people with disabilities, workplace social support is associated with lower levels of job anxiety [26] and facilitates return to work following a disability-related absence [27]. Note that neither of these studies sample Veterans; determining the extent to which these rehabilitation-relevant findings generalize to Veterans would be important.

The purpose of this study is to examine the role of workplace social support in job satisfaction among employed Veterans who manage PTSD. This research is critical to determine if the relationships found in the general population and other rehabilitation samples generalize to Veterans managing PTSD; if so, this could open new mechanisms for improvement of vocational rehabilitation outcomes.

Hypothesis 1: Assuming that workplace social support functions as an additional job resource, we expect that Veterans with higher levels of perceived workplace social support will report higher levels of job satisfaction.

Hypothesis 2: Based on the idea that managing PTSD functions as an additional job demand, we hypothesize that Veterans with more severe PTSD symptoms will report lower levels of job satisfaction after the variance attributable to workplace social support is controlled.

Hypothesis 3: The impact of workplace social support on job satisfaction will be moderated by severity of PTSD symptoms.

Hypothesis 4: Veterans reporting higher levels of individual social support domains (Career Mentoring, Coaching, Collegial Support, and Task Support) will report higher levels of job satisfaction after the variance attributable to PTSD symptoms is controlled.

## Methods

### Participants and procedure

Participants were 172 Veterans receiving outpatient services in an outpatient PTSD specialty clinic at a large Midwestern Veterans Affairs Medical Center. To receive services at this clinic, Veterans would have to have been evaluated by a licensed mental health provider using the Clinician Administered PTSD scale; individuals who do not meet criteria for a diagnosis of PTSD or subthreshold PTSD are referred to different clinics in the same medical center. Therefore, based on sampling procedure, it is assumed that all participants at one time met criteria for PTSD and were in continued treatment for trauma-related symptoms. All patients who presented for their clinic appointments were asked by clinic clerks to complete a brief anonymous questionnaire assessing current symptoms and employment status. Veterans were not compensated for participating in the survey. All participants received a consent form and cover letter. The consent form explained the risks associated with participation, and informed participants that participation was voluntary and that completion of the questionnaire was considered to be informed consent. All questionnaires were completed in the clinic waiting area. Questionnaires took approximately 15 minutes to complete. This study was approved and overseen by the Minneapolis VA Health Care System Human Studies Subcommittee.

### Instruments

#### PTSD checklist

The 17-item PTSD Checklist—Military version (PCL-M) [28] was used to assess past-month subjective PTSD symptom severity based on DSM-IV criteria. Among samples of veterans and non-veterans, Cronbach’s alpha for the PCL-M total score has ranged from .96^23^ to .94^24^. Consistent with these samples, alpha for the present sample was .94. Validity is based on positive, significant correlations with other validated instruments measuring PTSD, including both self-report and clinical interview instruments [29].

#### Mentoring and Communication Support Scale (MCSS)

The 15-item Mentoring and Communication Support Scale (MCSS; [30]) measures access to social support in the workplace. The instrument can be interpreted as a single scale measure of workplace social support, or as four separate subscales (Career Mentoring, Coaching, Collegial Support, and Task Support). Cronbach’s alpha for the total score in this sample was .91. Previous research has linked this scale with both job satisfaction and job tenure [21]. While the instrument was designed for employees in higher education settings, it has proven valid in studies of employees in a wide variety of organizations [21, 31]. Higher scores on this instrument are associated with poorer workplace social support; for ease of interpretation, it was reverse scored for this analysis, so that higher values corresponded with higher levels of workplace social support. In this sample, coefficient alphas for the subscales were .78 for Career Mentoring, .84 for Coaching, .87 for Collegial Support, and .87 for Task Support.

#### Job In General (JIG)

The 18-item adjective-checklist JIG [32] is a widely used global index of job satisfaction. Cronbach’s alpha for the instrument in this sample was .90, consistent with previous studies with alpha’s ranging from .91 to .95 [32]. Correlations with other measures of job satisfaction range from .67-.80 [32].

## Results

### Sample description

A total of 172 Veterans completed the survey; the outpatient PTSD clinic served approximately 575 Veterans during the time this data was collected, so the return rate was approximately 30%. The variables of interest for this study were only relevant to the 63 participants who were employed at the time of the survey, so only those 63 Veterans are included in the analysis. The sample was overwhelmingly male (84.40%) and Caucasian (93.80%). The mean age of the sample was 43.52 years old (15.15). Sixty-nine percent were employed full-time, and 30% reported that they were employed part-time. The modal monthly income category endorsed was $2001-$3000, and the modal level of education was “Some College.” The mean PCL-M score was 37.30 (14.85), with 67% of the sample obtaining a PCL-M score above a cutoff of 50 for probable PTSD. The mean MCSS total score was 43.59 (10.69) and the mean JIG score was 39.46 (11.22). Data on the types of trauma to which participants were exposed is not available.

### Hypothesis testing

Hypotheses were evaluated with an alpha of .03 to reduce experiment-wise Type I error inflation. Sidak-class method was used, rather than Bonferroni, Hochberg, or Holm, because in this small, preliminary, correlational study, inappropriate rejection of a valid hypothesis risks failure to pursue potentially fruitful research directions due to inadequate power, while inappropriate acceptance of an invalid hypothesis at this early point in this program of research does not risk in appropriate use of clinical interventions; because of this, a less-restrictive method of controlling Type I error is appropriate [33]. Intercorrelations of PTSD symptoms, JIG scores, and mentoring and communication support are presented in Table 1. Hypothesis tests are detailed in Tables 2 and 3. Hypotheses 1, 2, and 3 were tested using a single, hierarchical regression analysis, using JIG scores as the criterion variable. All predictor variables were centered (z-transformed) to limit the potential influence of multicollinearity and make direction of derived relationships and interactions easier to interpret. Total MCSS scores were entered in Step 1. PCL-M scores were entered in Step 2. The interaction of PCL-M scores and total MCSS scores were entered in Step 3. In Step 1, the model as a whole was statistically significant (F = 13.94, p < .001); workplace social support emerged as a statistically significant, positive predictor of job satisfaction (β = 0.44, p < .001) with an effect size of (R^2^ = .19). In Step 2, the model as a whole was statistically significant (F = 9.24, p < .001). In this step, workplace social support remained statistically significant (β = 0 .41, p = .001), but PTSD symptoms were not a significant predictor (β = -.23, p = .05). In Step 3, the model as a whole was statistically significant (F = 6.60, p = .001), but the interaction of PTSD symptoms and workplace social support was not statistically significant (β = -.13, p = .27). Hypothesis 1 was supported; workplace social support predicted higher levels of job satisfaction. Hypothesis 2 was not supported; after accounting for the variance due to workplace social support, PTSD symptoms did not predict job satisfaction; note that limited power and the alpha set for this study may be factors in that finding. Hypothesis 3 was not supported; the interaction of PTSD symptoms and workplace social support was not a significant predictor of job satisfaction, so the effect of workplace social support does not appear to be moderated by PTSD symptoms; both variables appear to be operating independently in predicting job satisfaction. Again, note that limited power and the alpha set for this study may be factors in this finding. To assess the possibility that multicollinearity may have distorted findings, we computed both tolerance and the Variance Inflation Factor (VIF). Tolerance ranged from .93–1.00, and VIF ranged from 1.02 to 1.08; all of these are in ranges that are not generally considered indicative of problematic multicollinearity[34].

**Table 1:** Intercorrelations, means, standard deviations and coefficient alphas.

	JIG	Career Mentoring	Coaching	Collegial Support	Task Support	Mean	Standard Deviation	α
PCL	-.29*	.34**	-.05	.04	.12	37.30	14.85	.94
JIG		.36**	.19	.30*	.46**	39.46	11.22	.90
Career Mentoring			.42**	.51**	.60**	12.49	3.39	.78
Coaching				.30*	.32*	9.56	2.73	.84
Collegial Support					.75**	11.54	3.74	.87
Task Support						9.98	3.61	.87

PCL = PTSD Checklist, JIG = Job in General (Job Satisfaction)

*p < .05,

**p < .01

**Table 2:** Multiple regression analyses for hypotheses 1–3: Criterion variable for all analyses is Job in General (job satisfaction).

Step/Predictor	B	SEB	β	Adj. R^2^	p	Observed Power
Step 1				0.18	<.001	
MCSS	.44	1.34	.44		<.001	.94
Step 2				0.22	<.001	
MCSS	4.62	1.32	.41		.001	.94
PCL	-2.59	1.32	-.23		.05	.49
Step 3				0.23	.001	
MCSS	4.36	1.33	-.39		.002	.06
PCL	-2.94	1.36	-.26		.034	.09
PCLxMCSS	-1.56	1.40	-.13		.27	.20

PCL = PTSD Checklist, MCSS = Total Score on the Mentoring and Communication Support Scale, PCLxMCSS = Interaction of PCL and MCSS

**Table 3:** Regression analyses for hypothesis 4: Criterion variable for all analyses is Job in General (job satisfaction).

Step/Predictor	B	SEB	β	Adj. R^2^	p	Observed Power
Step 1				.07	.025	
PCL	-0.22	0.09	-0.27		.025	.62
Step 2				.14	.005	
PCL	-0.14	0.09	-0.18		.160	.29
**Career Mentoring**	1.05	0.43	0.32		.017	.68

PCL = PTSD Checklist

Hypothesis 4 was tested using a series of four hierarchical multiple regression analyses predicting job satisfaction, again entering PTSD symptoms in step 1, and each of the individual subscales of the MCSS (Career Mentoring, Coaching, Collegial Support, and Task Support) in separate analyses for step 2 (See Tables 3–6). When given the statistical power benefit of being entered first in the regression equation, PTSD symptoms were a significant, negative predictor of job satisfaction (β = -0.29, p = .025). In the first regression, Career Mentoring emerged as a significant, positive predictor of job satisfaction (β = 1.05, p = .017; R^2^Δ = .09). In the second regression, Coaching failed to predict job satisfaction (β = 0 .88, p = .085; R^2^Δ = .05). In the third regression, Collegial Support emerged as a significant positive predictor of job satisfaction (β = 0.89, p = .016; R^2^Δ = .09). In the final regression, Task Support emerged as a significant, positive predictor of job satisfaction (β = 1.43, p < .001; R^2^Δ = .21). Hypothesis 4 was partially supported; Career Mentoring, Collegial Support, and Task Support positively predicted job satisfaction, but Coaching did not predict job satisfaction.

**Table 4:** Regression analyses for hypothesis 4: Criterion variable for all analyses is Job in General (job satisfaction).

Step/Predictor	B	SEB	β	Adj. R^2^	p	Observed Power
Step 1				.07	.025	
PCL	-0.22	0.09	-0.27		.025	.62
Step 2				.10	.018	
PCL	-0.23	0.09	-0.30		.018	.67
**Coaching**	0.88	0.50	0.21		.085	.41

PCL = PTSD Checklist

**Table 5:** Regression analyses for hypothesis 4: Criterion variable for all analyses is Job in General (job satisfaction).

Step/Predictor	B	SEB	β	Adj. R^2^	p	Observed Power
Step 1				.07	.025	
PCL	-0.22	0.09	-0.27		.025	.62
Step 2				.27	< .001	
PCL	-0.18	0.09	-0.24		.039	.63
**Task Support**	1.43	0.35	0.46		< .001	.69

PCL = PTSD Checklist

**Table 6:** Regression analyses for hypothesis 4: Criterion variable for all analyses is Job in General (job satisfaction).

Step/Predictor	B	SEB	β	Adj. R^2^	p	Observed Power
Step 1				.07	.025	
PCL	-0.22	0.09	-0.27		.025	.62
Step 2				.15	.004	
PCL	-0.21	0.09	-0.28		.024	.55
**Collegial Support**	0.89	0.36	0.30		.016	.98

PCL = PTSD Checklist

## Discussion

Limitations of this study include the small sample size, a single time point correlational design, and a treatment-seeking sample. Note especially that with this small sample size, power for the analysis of the interaction was especially low, and it is very possible that the moderation hypothesis is valid, but this study lacks the power to confirm it. Similarly, in Steps 2 and 3, PCL scores failed to emerge as significant predictors of job satisfaction, but in both cases, estimated power (.49 in Step 2 and .09 in Step 3) was low, and because the analysis involved multiple tests, our alpha was set at .03. Note that less stringent alphas would have resulted in a different interpretation of the results. These results may not generalize to Veterans with PTSD symptoms who are not in treatment, or who require more acute treatment than that provided by the outpatient clinic studied here. The mean score on the PCL-M in this sample was below the cutoff for a full PTSD diagnosis, so further studies would be necessary to generalize these findings across a broader spectrum of PTSD severity. The instrument used to measure PTSD symptoms was based on DSM-IV criteria, and was specific to military trauma. Further studies may be necessary to determine if findings still hold for DSM-5 criteria for PTSD, and for PTSD related to non-military trauma. Note as well that depression and other types of psychopathology may inflate scores on the PCL; given this limitation, further studies are necessary to determine if this pattern of findings is specific to PTSD or to psychological distress in general.

Further study of various dimensions of workplace social support would be a critical step in assessing the potential to use this as a modifiable variable in rehabilitation services. Note, for example, that in this sample, coaching was not associated with job satisfaction. Further research in the most effective dimensions of social support determining vocational adjustment would be indicated to adapt vocational rehabilitation interventions for optimal effectiveness. Furthermore, longitudinal study would be necessary to ascertain direction of causation. While our data support the idea that workplace social support facilitates job satisfaction, it is also possible that those who are more satisfied with their jobs also are more effective in eliciting social support. Most existing literature on relationships between workplace social support and vocational adjustment is cross-sectional, although one longitudinal study [22] found that workplace social support precedes organizational citizenship behaviors in a cross-lag analysis.

Despite these limitations, given the convergence of findings that workplace social support is associated with better vocational adjustment, along with this finding that this relationship generalizes to Veterans seeking treatment for PTSD, it appears that there is ample reason to explore the potential for increasing social support as a rehabilitation intervention. This area of inquiry is particularly promising given the large effect size found in this sample. Next steps in this line of research would require longitudinal studies of workplace social support, exploring multiple dimensions of both social support and vocational adjustment. Should the direction of causation be from workplace social support to vocational adjustment, such a study would identify the dimensions of workplace social support that might be targeted for intervention. Interventions may include a) training Veterans in social skills necessary to develop workplace social support, b) educating workplace supervisors about the most effective ways to support employees managing PTSD (for example, by providing career mentoring), and c) teaching Veterans’ work units how to provide effective collegial and task support within the team. Supplementing existing PTSD vocational rehabilitation programming with interventions to enhance workplace social support may increase resources not readily available to Veterans with PTSD, and thus improve vocational rehabilitation outcomes. Results also suggest potential utility for further research exploring the extent to which enhanced workplace social support may improve outcomes among veterans with other types of mental health challenges.

These types of workplace enhancement rehabilitation interventions are consistent with the Supported Employment model widely used in VA, which uses onsite job coaching and a service model integrating the employer, job coach, and mental health providers. Supported Employment (SE) job coaches consistently facilitate development of natural supports in the work place; developing additional interventions to expand the social support resources in the workplace would constitute a natural extension of this model, and may improve outcomes in the job maintenance phase of SE services.

## Conclusions

Hypothesis 1 was supported; Veterans with more workplace social support reported higher levels of job satisfaction, accounting for 19% of the variance. Hypothesis 2 was not supported. After controlling for workplace social support, PTSD symptom severity was not a statistically significant predictor of job satisfaction. Hypothesis 3 was not supported; there is not evidence that PTSD symptom severity moderates the relationship between workplace social support on job satisfaction, although limited power may be a factor in this finding. Hypothesis 4 was partially supported; Veterans reporting higher levels of Career Mentoring, Collegial Support, and Task Support reported higher levels of job satisfaction. It was notable that social support accounted for more variance in job satisfaction than PTSD symptoms. The direction of relationships found is consistent with the premise that managing PTSD symptoms is a significant job demand, while workplace social support constitutes an effective job resource. In this sample, career mentoring, collegial support, and task support predicted higher levels of job satisfaction, while coaching did not. De-identified data is available via e-mail request to the first author.

## Supporting information

S1 File Supporting information. Minimal dataset underlying Tables 1–6.
(XLSX)
